# Continuous or interrupted suture technique for hepaticojejunostomy during pancreatoduodenectomy (HEKTIK trial): study protocol of a randomized controlled multicenter trial

**DOI:** 10.1186/s13063-022-06427-1

**Published:** 2022-06-06

**Authors:** Maximilian Brunner, Henriette Golcher, Christian Krautz, Stephan Kersting, Georg F. Weber, Robert Grützmann

**Affiliations:** 1 Department of General Surgery, University Hospital of Friedrich-Alexander-University, Krankenhausstraße 12, Erlangen, Germany; 2 Department of General Surgery, University Hospital Greifswald, Ferdinand-Sauerbruch-Straße, Greifswald, Germany

**Keywords:** Hepaticojejunostomy, Suture technique, Pancreatoduodenectomy, Continuous suture technique, Interrupted suture technique

## Abstract

**Background:**

Hepaticojejunostomy is commonly performed in hepato-bilio-pancreatic surgery, particularly during pancreaticoduodenectomy. The purpose of this study is to evaluate the safety and efficiency of two commonly applied suture techniques (the interrupted versus the continuous suture technique) in patients undergoing a hepaticojejunostomy during pancreatoduodenectomy.

**Methods:**

The HEKTIK trial is a multicenter, randomized controlled, patient-blinded surgical explorative trial with two parallel study groups. An adaptive sample size design was chosen: First, 100 patients scheduled for surgery including a hepaticojejunostomy will be randomized 1:1 either to the interrupted suture technique or the continuous suture technique after informed consent. Based on this data, needed sample size will be adjusted.

The primary endpoint will be the occurrence of anastomotic leakage of hepaticojejunostomy, defined as bilirubin concentration in the drain fluid at least 3 times the serum bilirubin concentration on or after postoperative day 3 or as the need for radiologic or operative intervention resulting from biliary collections or bile peritonitis (according to the definition of ISGLS). Further perioperative parameters like other morbidities as well as duration and costs of the hepaticojejunostomy will be analyzed as secondary outcomes.

**Discussion:**

Until now there are no randomized controlled comparative data of these two suture techniques for hepaticojejunostomy. The HEKTIK trial will investigate the yet unanswered question of whether the interrupted suture or the continuous suture technique has advantages performing a hepaticojejunostomy during pancreatoduodenectomy.

**Trial registration:**

German Clinical Trials Register DRKS00024395. Registered on 01 February 2021.

**Supplementary Information:**

The online version contains supplementary material available at 10.1186/s13063-022-06427-1.

## Background

Hepaticojejunostomies represent a common step during pancreatic and liver surgery including partial pancreatoduodenectomy, major liver resections, and bile duct resections. Overall, a hepaticojejunostomy is considered as a safe procedure with low rates of postoperative complications including a leakage rate of 2.3% to 5.6% [1, 2]. However, failure of this anastomosis leads to considerable consequences with a high risk of prolonged hospitalization and the need for interventional drainage or re-laparotomy, which is associated with high morbidity and mortality, even in high volume centers [3, 4]. Known risk factors for leakage are previous chemoradiation, preoperative biliary drainage, impaired liver synthesis capacity, malnutrition, obesity, and anastomosis at the level of hepatic duct bifurcation [1].

However, leakage of a hepaticojejunostomy could always be associated with the surgical technique used. There are especially two common techniques used for hepaticojejunostomy: the interrupted suture technique and the continuous suture technique. According to a current national survey in Germany 56% of the participating hospitals stated that they use both techniques for performing a hepaticojejunostomy depending on the surgical sites, while 40% of the hospitals adhere always to one of the mentioned two techniques. These results show a relevant heterogeneity of the techniques used among hospitals in Germany [5]. There is an ongoing debate between advocates of both techniques, who argue with universal use (also for small ducts) and a potentially lower stenosis rate for the interrupted suture technique and with a better sealing of the anastomosis and operative time saving for the continuous suture technique.

Despite the frequent necessity of hepaticojejunostomies in surgery and the relevant consequences for the patient with leakage, there are no randomized studies to compare the different surgical techniques. For this reason, the presented randomized controlled multicenter trial was initiated to assess whether there is a significant difference in the occurrence of anastomotic leakage between the two groups interrupted suture versus continuous suture technique for performance of a hepaticojejunostomy during pancreatoduodenectomy.

## Methods/design

### Administrative information

The trial was initiated by the surgical department of the university hospital Erlangen, Friedrich-Alexander-university Erlangen-Nuremberg. The study protocol was created in 04/2019 (version 1.0) and is still valid. The coordinators/sponsors of the trial are the first and both senior authors of this study protocol. The heads of this trial are primarily responsible for all study matters (planning, implementation, management, analysis, publication) and can be contacted as follows: Krankenhausstraße 12, 91054 Erlangen, Germany; phone: +49 9131 85 33296. The trial was registered at the German Clinical Trials Register (DRKS00024395) on February 01, 2021. The central organization costs are funded by internal clinic funds of the Department of general surgery, university hospital of Friedrich-Alexander-University, Erlangen and by the “Verein zur Förderung des Tumorzentrums der Universität Erlangen-Nürnberg e.V.”. There is no external funding support of this trial outside the university hospital Erlangen.

#### Protocol amendments

The study protocol was adapted once as part of revision for this publication (version 2.0).

### Trial design and study setting

The objective of the HEKTIK trial is to investigate whether there is a significant difference in the occurrence of anastomotic leakage between the two groups interrupted suture versus continuous suture technique for performance of a hepaticojejunostomy during pancreatoduodenectomy. The study is designed as a randomized controlled, national (Germany), patient-blinded multicenter trial with the performance of a hepaticojejunostomy in interrupted suture technique in one arm and in continuous suture technique in the other arm. To homogenize patient collective and the risk profile for occurrence of anastomotic leakage of hepaticojejunostomy only patients with pancreatoduodenectomy were included. Until now, there is only the institution of the study coordinators, that recruits patients for the HEKTIK trial. Further high-volume centers are planned to initiate soon.

### Aim of the study and study endpoints

Primary endpoint of this trial is the prevelance of an anastomotic leakage of the hepaticojejunostomy, as it was defined by the International Study Group of Liver Surgery (ISGLS) in 2011 [6]:

*“After evaluation of the postoperative course of bilirubin levels in the drain fluid of patients who underwent hepatobiliary and pancreatic operations, bile leakage was defined*

1. *as bilirubin concentration in the drain fluid at least 3 times the serum bilirubin concentration on or after postoperative day 3*
  *or*
2. *as the need for radiologic or operative intervention resulting from biliary collections or bile peritonitis.”* [6]

The secondary endpoints are:

- Total operative time
- Postoperative increase in cholestasis parameters (γGT, alkaline phosphatase, bilirubin)
- Reoperation rate (during hospital stay and at 3 and 12 months postoperative)
- Reintervention rate (e.g., ERCP, PTCD) (during hospital stay and at 3 and 12 months postoperative)
- Morbidity (during hospital stay and at 3 and 12 months postoperative)
  The recorded morbidity includes:
  - *Clavien-Dindo classification:* see Table 1 [7].
  - *Comprehensive complications index (CCI):* The CCI is calculated as the sum of all complications that are weighted for their severity (multiplication of the median reference values from patients and physicians). The final formula yields a continuous scale to rank the severity of any combination of complications from 0 to 100 in a single patient [8]
  - *Postoperative pancreatic fistula (POPF):* defined as a drain output of any measurable volume of fluid with an amylase level > 3 times the upper limit of institutional normal serum amylase activity, associated with a clinically relevant development/condition related directly to the postoperative pancreatic fistula [9]
- Mortality (during hospital stay and at 3 and 12 months postoperative)
- Duration of the hepaticojejunostomy performance
- Material costs for the hepaticojejunostomy
- Stenosis rate of hepaticojejunostomy at 3 and 12 months postoperative

**Table 1:** Clavien-Dindo classification

Grade	Definition
Grade I	Any deviation from the normal postoperative course without the need for pharmacological treatment or surgical, endoscopic, and radiological interventions. Allowed therapeutic regimens are drugs such as antiemetics, antipyretics, analgesics, diuretics, electrolytes, and physiotherapy. This grade also includes wound infections opened at the bedside.
Grad II	Requiring pharmacological treatment with drugs other than such allowed for grade I complications Blood transfusions and total parenteral nutrition are also included
Grade III	Requiring surgical, endoscopic or radiological intervention
Grade IIIa	Intervention not under general anesthesia
Grad IIIb	Intervention under general anesthesia
Grade IV	Life-threatening complication requiring IC/ICU management
Grade IVa	Single organ dysfunction (including dialysis)
Grade IVb	Multiorgan dysfunction
Grade V	Death of patient

### Study population (inclusion and exclusion criteria)

**Fig. 1:**
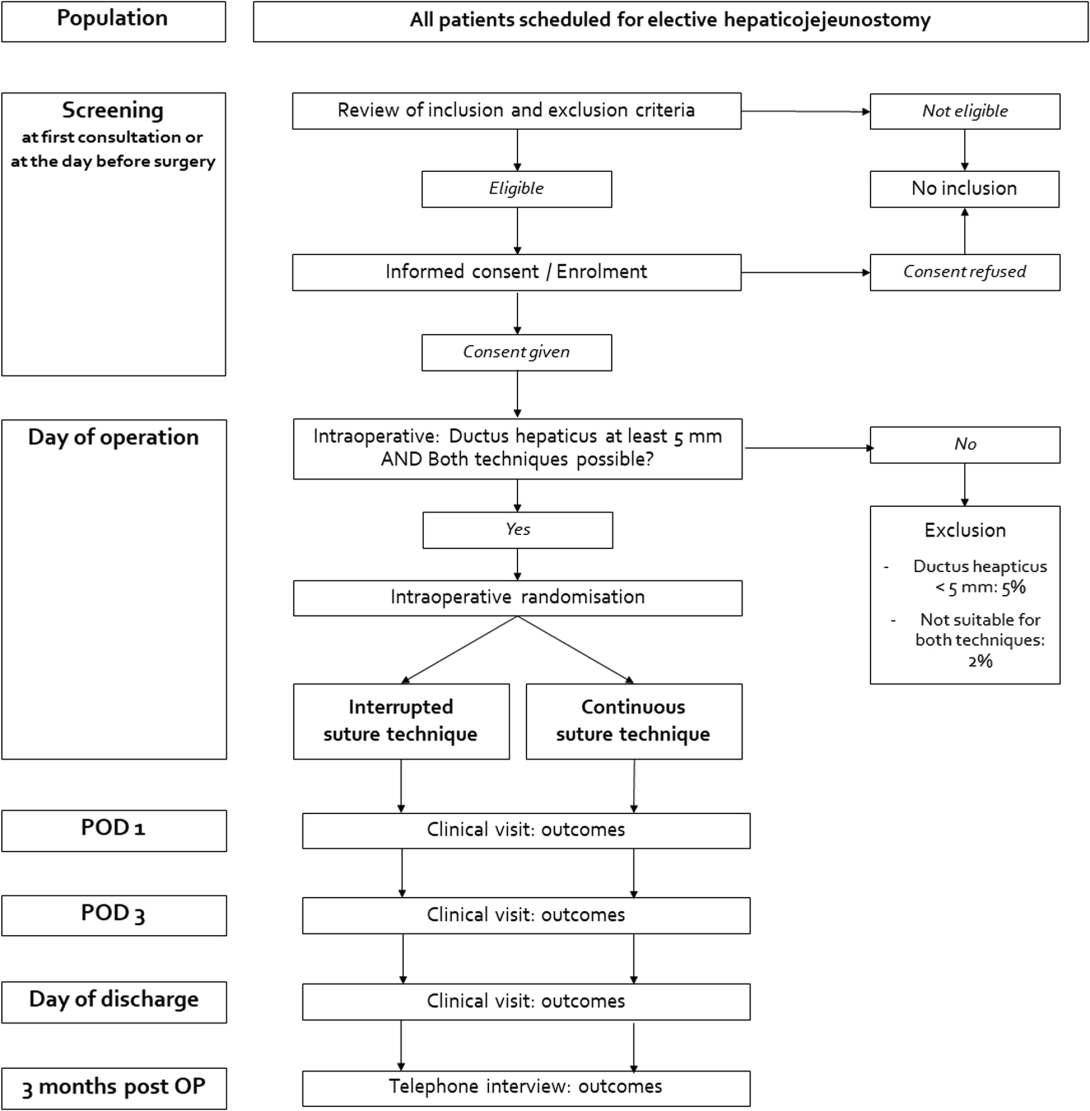
Flow chart of the HEKTIK trial. POD, postoperative day

All patients scheduled for elective hepaticojejeunostomy during pancreatoduodenectomy will be screened consecutively for eligibility and will be informed about the HEKTIK trial. The diameter of the ductus hepaticus be anastomosed must be at least 5 mm, which is determined by measuring the ductus hepaticus intraoperatively. There are the following further general inclusion criteria: age equal to or older than 18 years, American Society of Anesthesiologists (ASA) score I–III, and a completed written informed consent form (Fig. 1).

### Patient withdrawal criteria

Patients are free to stop their trial participation at any time and without giving reasons for their decision. When a trial participant withdraws his/her informed consent, he/she is asked to decide whether his/her data captured so far may be analyzed or if it should be discarded. In addition, if intraoperatively the diameter of the ductus hepaticus is less than 5 mm or in the surgeon’s opinion there is only one possible technique or one better technique for the patient, the trial participation will be stopped for this patient (Fig. 1). In this case, the patient will not be randomized, and the reason for screening failure must be recorded in the screening log. All randomized patients, including those with premature trial termination, will be included in the final analysis.

### Surgical technique

The hepaticojejunostomy will be performed as a part of pancreatoduodenectomy and can be placed on the same jejunal loop as the pancreatojejunostomy or on a second jejunal loop.

The common preparation before suturing the hepaticojunostomy includes an opening of the hepatic duct with corner sutures and an antimesenterical jejunal incision. After the corner sutures are placed at the hepatic duct, the diameter of the ductus hepaticus is measured in mm and documented in the CRF.

The further procedure depends on the randomized technique:

1. Interrupted suture technique: with this technique both the posterior and the anterior walls are performed single-rowed using interrupted stitches.
2. Continuous suture technique: with this technique both the posterior and the anterior walls are performed single-rowed using continuous sutures.

There are no specifications for the suture material and the number of sutures used, but these data will be documented in the CRF. If an anastomotic leakage is detected intraoperatively, any number of interrupted sutures may be added to the hepaticojejunostomy. The measurement of the duration of the hepaticojeunostomy begins with the first suture (excluding the corner sutures of the hepatic duct) and ends with the cutting of the last thread, which is necessary to complete the hepaticojeunostomy. The application of an abdominal drainage near the hepaticojejunostomy is standard in our department and is recommended, but not mandatory.

Used suture material, number of sutures, number of interrupted stiches, number and place of abdominal drains, and the time for hepaticojejunostomy are documented in the CRF. In addition, the surgeon’s experience performing hepaticojejunostomies is recorded and documented in the CRF.

### Randomization and blinding

After the inclusion criteria have been confirmed and the patient's consent has been obtained preoperatively by trained medical staff as well as exclusion criteria (e.g., no performance of a hepaticojejunostomy due to irresectability of the pancreatic tumor) have been excluded intraoperatively, randomization will be carried out intraoperatively immediately before the hepaticojejunostomy is performed. Randomization only takes place if the surgeon considers the present situs to be suitable for both suturing techniques and the ductus hepaticus is at least 5 mm (Fig. 1).

#### Sequence generation

The randomization is designed with fixed block sizes in a 1:1 allocation ratio and is generated using research randomizer (www.randomizer.org). The generation of randomization codes and the randomization on the day of the surgery is performed independently of the surgical team by authorized study personnel only. More details of the randomization will be kept safe and confidential as long as this trial is ongoing.

#### Allocation concealment mechanism

Ass allocation concealment mechanism, the authorized study personnel, who perform randomization, are not involved in the inclusion of patients as well as in the treatment part.

#### Blinding

To reduce bias blinding of study contributors is a common and effective measure [10]. However, in our study, only patients were blinded to the study treatment. Surgeons cannot be blinded as they perform the study therapy. The data analyst, data collector, and the outcome assessor were not blinded due to the robust outcome parameters.

### Study visits and data collection

This trial consists of a total of seven study visits, which take place at the following times and include the following milestones of the trial (Table 2):

- Preoperative at the first consultation or at the day before surgery: verification of all eligibility criteria (inclusion and exclusion criteria), obtaining patient informed consent, completing the screening form including laboratory results
- Day of operation: randomization, performing hepaticojejunostomy in the randomized surgical technique, completing the surgical form
- Postoperative day 1 (POD 1): completing the morbidity and mortality form including laboratory results and an intraabdominal drainage analysis of bilirubin
- Postoperative day 3 (POD 3): completing the morbidity and mortality form including laboratory results and an intraabdominal drainage analysis of bilirubin
- Day of discharge: completing the discharge form including the morbidity and mortality form
- Three and 12 months after operation: completing the morbidity and mortality form by telephone interview

**Table 2:** Visit schedule of the HEKTIK trial

	Study period						
	Screening at first consultation or at the day before surgery	Day of operation	POD 1	POD 3	Day of discharge	3 months after operation	12 months after operation
In-/exclusion criteria	X						
Patient informed consent	X						
Screening form	X						
Laboratory tests	X		X	X			
Randomization		X					
Used surgical technique		X					
Surgical form		X					
Morbidity and mortality form			X	X	X	X	X
Drainage analysis			X	X			
Discharge form					X		
Telephone interview						X	X

Preoperative and postoperative stationary data are collected by the investigators, operative data by the operating surgeon and the data at three months postoperative by authorized study personnel.

The screening form contains demographic data including basis data, disease course, and comorbidities as well as preoperative blood results (including hemoglobin, white blood cells, creatinine, albumin, serum c-reactive protein, and liver enzymes). In the surgical form data about the operation like duration and kind of surgery as well as data about the hepaticojejunostomy (duration, suture material, information about the hepatic duct) is recorded. The postoperative forms include always the documentation of morbidity and mortality as well as at POD 1 and 3 laboratory results (including hemoglobin, white blood cells, creatinine, albumin, serum c-reactive protein, Quick, bilirubin as well as liver enzymes and cholestasis parameters) and the bilirubin concentration in the abdominal drain being close to the hepaticojejunostomy. At discharge histological results and length of hospital stay are additionally documented.

### Post-trial care

In addition to the two calls after 3 and 12 months postoperatively to record morbidity, there is no specific follow-up program for the included patients. Further follow-up care depends in particular on the underlying disease: e.g. if pancreatic carcinoma is present, we would offer a quarterly follow-up check-up in the first year and after that a follow-up every 6 months.

### Documentation and data management

The collected data is documented using a case report form (CRF) with pseudonymization of patients. For data protection, the CRF contains only an individual identification code including a center number and a patient number. All CRFs are reviewed by the investigators. All data collected is treated with strict confidentiality in accordance with the data protection and the GCP guidelines. Access to the data is only permitted to authorized personnel.

### Data monitoring and quality assurance

Data monitoring and quality assurance are guaranteed by an inspection of all CRFs together with other documents such as the surgery report and the discharge letter. Therefore there is no data monitoring committee (DMC) as well as no data monitoring visits, as the risk of study bias through missing extended monitoring is considered low. There is no mandatory intraoperative photo documentation of the hepaticojejunostomy. Since there are only limited technical requirements for the performance of the hepaticojejunostomy, the risk of deviations from the study protocol should be low. In addition, the anatomical and surgical characteristics (such as the number and type of suture material, nature of the hepatic duct) are recorded in the CRF.

The trial can be stopped by the coordinators of the trial in case of reasons recommending termination of the trial like medical or ethical rationales as well as inadequate patient recruitment or additional external evidence.

### Assessment of safety

Any deviations from the normal postoperative course will be documented in a special morbidity form during the whole study period (from signature of the informed consent until 12 months postoperative) and classified according to the Clavien-Dindo classification. The morbidity form record the symptoms, the diagnostics and therapy taken and the beginning, severity, duration, consequences, and outcome of the event as well as to what extent a causality with the trial intervention is possible.

A major morbidity event (Clavien-Dindo ≥ 3) must be reported to the sponsor by the responsible investigator within 24 hours after their occurrence. The heads of this trial are responsible for the assessment of these events and the possible need to notify the responsible ethics committee.

### Ethical aspects

The trial is to be conducted in line with the Declaration of Helsinki. All trial documents (study protocol, CRF, patient information, informed consent) were approved by the ethical committee of the Friedrich-Alexander-university Erlangen-Nuremberg (number: 167_19 B; decision on 22.05.2019). All screened patients will be informed in detail about the study and possible risks. All further participating centers have to obtain approval from their local ethics committee.

### Statistical considerations and sample size calculation

Literature was systematically reviewed to identify all published data to this topic (see Supplementary Fig. 1). There were no randomized controlled trials and only one retrospective analysis from Japan, which compared the interrupted suture technique with the continuous suture technique in the setting of liver transplantations [11]. However, these data are not suitable for a meaningful sample size calculation, as the leakage rates of this reported trial come from a different setting and seem to be high compared to data derived mainly from hepaticojejunostomies only or as part of a pancreatoduodenectomy (5.6% using interrupted sutures [1] and 2.3% using both techniques [2], respectively).

Therefore, an adaptive sequential sample size design was chosen: The sample size per patient group was set exploratively at a group size of 50 patients. Total sample size was 100 patients. The prevalence of the primary endpoint in the 100 patients examined then forms the data basis for an adequate further sample size analysis and consequently for a sample size adjustment. To proceed the study with the then new calculated sample size, some criteria have to be met: First, the absolute difference in the primary endpoint must be at least 1% to justify clinical relevance of the study. Second, the calculated sample size per group should not exceed 1000 patients in order to be able to meaningfully end the study.

Due to the design with multiple testing an error correction will be performed in statistical analysis. Due to the lack of reliable data in the literature, this adaptive design offers the best opportunity to get meaningful data with adequate power.

Statistical analysis will be performed using SPSS software. An intention-to-treat and a per-protocol-analysis will be used for the analysis of the primary outcome. Comparisons of metric and ordinal data will be calculated with the Student *t*-test or Mann-Whitney *U* test. The chi-square test was used for categorical data. Statistical significance will be set at *p* < 0.05.

### Dissemination policy

After completion of the study, the results will be published in an international journal.

## Discussion

This clinical trial is one of the first randomized controlled study to investigate the safety and efficiency of two commonly applied suture techniques (the interrupted versus the continuous suture technique) in patients undergoing a hepaticojejunostomy during pancreatoduodenectomy. In addition to this study, there could be identified a second ongoing registered study on the same issue in China (ChiCTR1900020605).

Although the performance of a hepaticojejunostomy is a common procedure in hepato-pancreato-biliary surgery and a leakage of this anastomosis is associated with relevant morbidity and mortality, very few studies have dealt with this topic to date. A comparison of the suturing techniques with regard to the leakage rates was only carried out in one retrospective study in the setting of liver transplantations. This illustrates the necessity and usefulness of a study to compare the suturing techniques for hepaticojejunostomy during pancreatoduodenectomy.

Before initiating the present trial, a Germany-wide survey was carried out on the application of the various suturing techniques for hepaticojejunostomies. This confirmed that there is enormous heterogeneity in the technology used. While the majority use both techniques depending on the intraoperative situs, 40% of those questioned are rigidly attached to their technique. The bile duct diameter was cited as the most important decision criterion for those who use both techniques. This shows that very small bile ducts in particular can often be better treated using the interrupted suture technique. Therefore, the lower limit of the bile duct diameter in our study was set to 5 mm.

Due to the small amount of data comparing surgical techniques for hepaticojejunostomies, the sample size calculation is a particular challenge. There are only data available comparing the interrupted and the continuous suture technique in the liver transplant setting, where the risk of leakage is likely to be higher. Published data from the pancreatic surgery setting show lower leakage rates. However, in this pancreatic setting, there are no comparative data. To compensate for this lack of data, an adaptive design with an interim analysis after 100 patients was incorporated. At this point in time, the sample size can be adjusted, which significantly increases the accuracy of the sample size calculation.

In summary, we have emphasized the importance of the suture technique for hepaticojejunostomies, as hepaticojejunostomies are common procedures and a leakage of the hepaticojejunostomy is associated with relevant sequelae for the patients. In addition, the paucity of the available data was presented by performing a systematic literature review. Therefore, the initiation and implementation of this trial represents the logical step.

## Trial status

Recruitment is ongoing (study protocol version 2.0). The first patient was enrolled in March 2020. Recruitment of the last patient is planned for March 2024.

## Supplementary Information

**Additional file 1: Sup. Figure 1.** Overview of the literature search for sample size calculcation.

**Additional file 2.**

## Data Availability

We ensure that our datasets will be either deposited in publicly available repositories or presented in the main manuscript or additional supporting files.

## References

[1] Antolovic D, Koch M, Galindo L, Wolff S, Music E, Kienle P, Schemmer P, Friess H, Schmidt J, Büchler MW, Weitz J (2007). Hepaticojejunostomy--analysis of risk factors for postoperative bile leaks and surgical complications. J Gastrointest Surg.

[2] de Castro SM, Kuhlmann KF, Busch OR, van Delden OM, Laméris JS, van Gulik TM, Obertop H, Gouma DJ (2005). Incidence and management of biliary leakage after hepaticojejunostomy. J Gastrointest Surg.

[3] Akamatsu N, Sugawara Y, Hashimoto D (2011). Biliary reconstruction, its complications and management of biliary complications after adult liver transplantation: a systematic review of the incidence, risk factors and outcome. Transpl Int.

[4] Chok KS, Ng KK, Poon RT (2009). Impact of postoperative complications on long-term outcome of curative resection for hepatocellular carcinoma. Br J Surg.

[5] Brunner M, Stockheim J, Krautz C, Raptis D, Kersting S, Weber GF, Grützmann R (2018). Continuous or interrupted suture technique for hepaticojejunostomy? A national survey. BMC Surg.

[6] Koch M, Garden OJ, Padbury R, Rahbari NN, Adam R, Capussotti L, Fan ST, Yokoyama Y, Crawford M, Makuuchi M, Christophi C, Banting S, Brooke-Smith M, Usatoff V, Nagino M, Maddern G, Hugh TJ, Vauthey JN, Greig P, Rees M, Nimura Y, Figueras J, DeMatteo RP, Büchler MW, Weitz J (2011). Bile leakage after hepatobiliary and pancreatic surgery: a definition and grading of severity by the International Study Group of Liver Surgery. Surgery.

[7] Dindo D, Demartines N, Clavien PA (2004). Classification of surgical complications: a new proposal with evaluation in a cohort of 6336 patients and results of a survey. Ann Surg.

[8] Slankamenac K, Graf R, Barkun J, Puhan MA, Clavien PA (2013). The comprehensive complication index: a novel continuous scale to measure surgical morbidity. Ann Surg.

[9] Bassi C, Marchegiani G, Dervenis C, Sarr M, Abu Hilal M, Adham M, Allen P, Andersson R, Asbun HJ, Besselink MG, Conlon K, Del Chiaro M, Falconi M, Fernandez-Cruz L, Fernandez-Del Castillo C, Fingerhut A, Friess H, Gouma DJ, Hackert T, Izbicki J, Lillemoe KD, Neoptolemos JP, Olah A, Schulick R, Shrikhande SV, Takada T, Takaori K, Traverso W, Vollmer CR, Wolfgang CL, Yeo CJ, Salvia R, Buchler M, International Study Group on Pancreatic Surgery (ISGPS) (2017). The 2016 update of the International Study Group (ISGPS) definition and grading of postoperative pancreatic fistula: 11 years after. Surgery.

[10] Probst P, Zaschke S, Heger P, Harnoss JC, Hüttner FJ, Mihaljevic AL, Knebel P, Diener MK (2019). Evidence-based recommendations for blinding in surgical trials. Langenbecks Arch Surg.

[11] Kasahara M, Egawa H, Takada Y, Oike F, Sakamoto S, Kiuchi T, Yazumi S, Shibata T, Tanaka K (2006). Biliary reconstruction in right lobe living-donor liver transplantation: comparison of different techniques in 321 recipients. Ann Surg.

