# Supplementary figures and images for: Continuous or interrupted suture technique for hepaticojejunostomy during pancreatoduodenectomy (HEKTIK trial): study protocol of a randomized controlled multicenter trial
Source: Trials. 2022 Jun 6;23:467. doi: 10.1186/s13063-022-06427-1 (PMC9169310; doi:10.1186/s13063-022-06427-1)

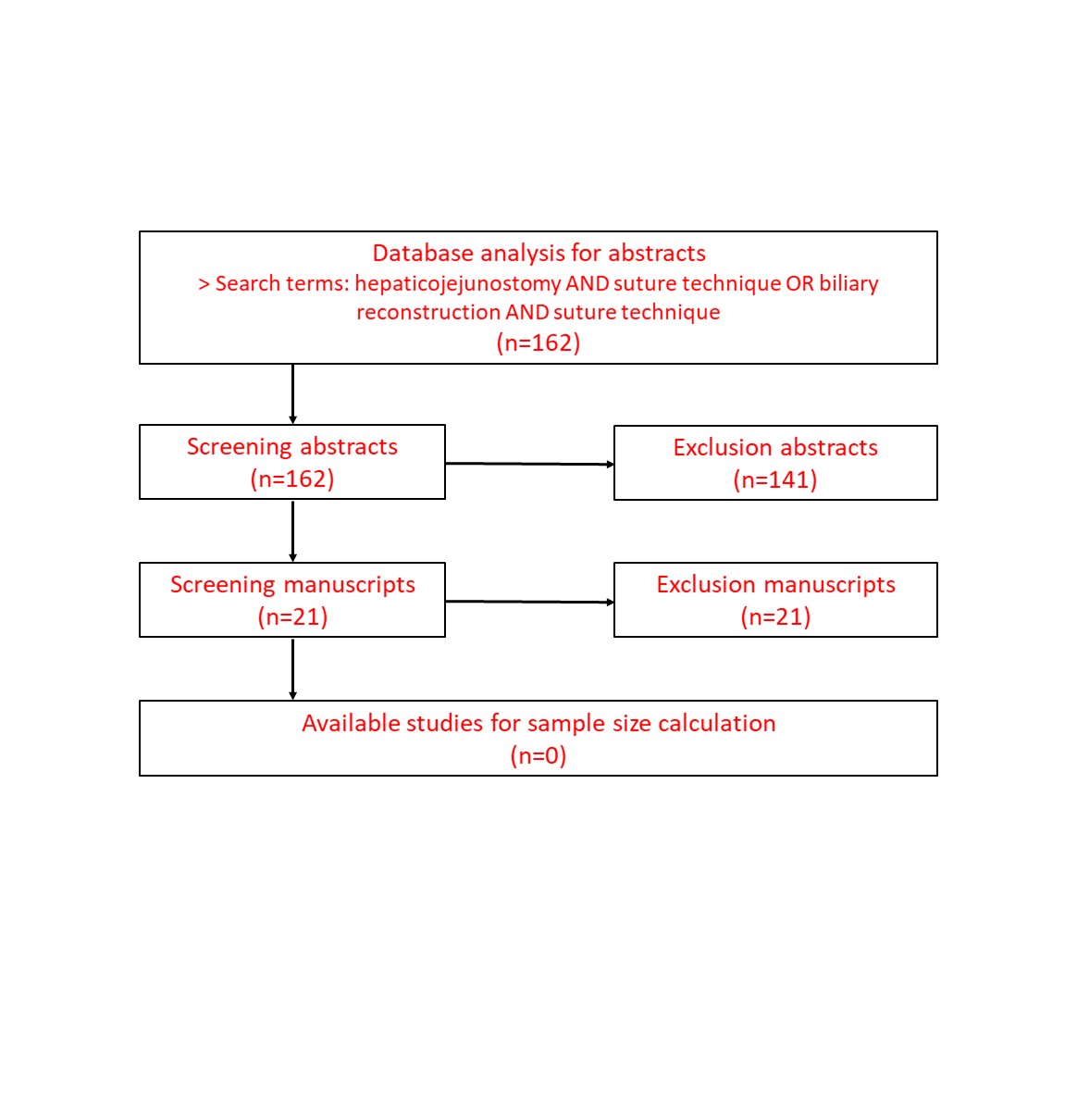

Supplement: Supplementary file 1 — Additional file 1: Sup. Figure 1. Overview of the literature search for sample size calculcation. [file 13063_2022_6427_MOESM1_ESM.jpg]
