# Supplementary material for: Continuous or interrupted suture technique for hepaticojejunostomy during pancreatoduodenectomy (HEKTIK trial): study protocol of a randomized controlled multicenter trial
Source: Trials. 2022 Jun 6;23:467. doi: 10.1186/s13063-022-06427-1 (PMC9169310; doi:10.1186/s13063-022-06427-1)
Supplement: Supplementary file 2 — Additional file 2. [file 13063_2022_6427_MOESM2_ESM.docx]

**Visit 1 – Screening (Page 1 / 4)**

Date of screening: **2 0** *(dd/mm/yyyy)*

Date of informed consent: **2 0** *(dd/mm/yyyy)*

| **Inclusion criteria no yes**   1. Age equal or greater than 18 years ⬜ ⬜ 2. Scheduled for elective open hepaticojejunostomy ⬜ ⬜   (ductus hepaticus communis + jejunum) during  pancreatoduodenectomy   1. ASA classification I-III ⬜ ⬜ 2. Written informed consent ⬜ ⬜ |
| --- |
| **Exclusion criteria no yes**   1. Hepaticojejunostomy performed ⬜ ⬜   as a own procedure or during another surgery  other than pancreatoduodenectomy |

**Visit 1 – Screening (Page 2 / 4)**

**Demographic data**

Date of birth: **1 9** *(dd/mm/yyyy)*

Age: years Gender: male female

Height: cm Weight: , kg

ASA: I II III IV

**Smoking habits**

Never smoked Previous smoker Current smoker Pack years: _______­­

**Preoperative biliary stenting**

No Yes If yes, please specify: ____________________

**Comorbidities**

Liver function: not impaired or Child-Pugh-Class A B C


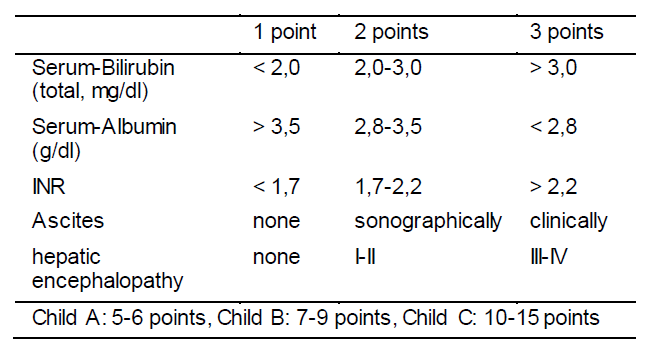


**No Yes**

Diabetes:

If yes, insulindependent?

Chronic renal insufficiency:

If yes, need of dialysis?

Coronary heart disease:

Arterial hypertension:

COPD:

**Visit 1 – Screening (Page 3 / 4)**

**Neoadjuvant therapy**  yes no

If yes, please specify: Chemotherapy

Radiotherapy

Both

Period: ________________ _______________

If radiation, dose of radiation: _______ Gy

If chemotherapy, chemotherapeutical agents:

____________________­­­­­­­­­­­­­­­­­­­_________________________________________

_____________________________________________________________

**Previous surgery**

Number of previous abdominal surgeries: ____________

Kind of surgeries:

**Preoperative diagnosis**

Pancreatic ductal adenocarcinoma Distal bile duct carcinoma

Pancreatic neuroendocrine neoplasm Duodenal carcinoma

IPMN or cystic pancreatic neoplasm Ampullary carcinoma

Benigne bile duct stenosis Chronic pancreatitis

Other: ____________________________________________________

**Visit 1 – Screening (Page 4 / 4)**

**Preoperative laboratory tests**

Date of blood sample taking **2 0** *(dd/mm/yyyy)*

Hemoglobin __________ (g/dl) Leucocytes __________ (cells/ml)

Creatinine __________ (mg/dl) Albumin __________ (g/l)

ASAT __________ (U/l) ALAT __________ (U/l)

AP __________ (U/l) GGT __________ (U/l)

Bilirubin __________ (mg/dl) CRP __________ (mg/dl)

Quick value __________ (%)

Name of investigator in block letter: ___________________________________

Date: _____________ Signature: ___________________________

**Visit 2 – Day of operation (Page 1 / 2)**

Date of operation **2 0** *(dd/mm/yyyy)*

**Operation**

Operating time: *(hh/mm)*

Kind of operation: _________________________________________

Surgeons experience: < 20 hepaticojejunostomies

20 – 50 hepaticojejunostomies

> 50 hepaticojejunostomies

**Operating details**

Duration of hepaticojejunostomy: *(mm/ss)*

Randomization in

- hepaticojejunostomy in interrupted suture technique: yes no

If yes, number of interupted stiches: ______________________

- hepaticojejunostomy in continuous suture technique: yes no

If yes, number of additional interrupted stiches: ____________________

Used suture material:

| Suture material | Number |
| --- | --- |
|  |  |
|  |  |
|  |  |

**Bile duct conditions**

Bile duct diameter: _________________________________

(measured in mm, after the corner sutures are placed at the hepatic duct)

Length of bile duct: long (>2cm) short (<2cm)

(from branching of ductus hepaticus)

Bile duct wall thickness: thick delicate

**Visit 2 – Day of operation (Page 2 / 2)**

Combined arterial resection: yes no

**Drains**

Number of inserted drains: _____________________

Drain placed at hepaticojejunostomy? yes no

Name of investigator in block letter: ___________________________________

Date: _____________ Signature: ___________________________

**Visit 3 – POD 1 (Page 1 / 3)**

Date of visit **2 0** *(dd/mm/yyyy)*

**Morbidity**

Has any complication occurred or yes no

is still active since the last visit?

**If yes, please specify and complete the AE / SAE form for each event separately!**

| **Complication** | **Diagnostic tests** | **Therapy** |
| --- | --- | --- |
| Postoperative yes no  insufficiency of  hepaticojejunostomy  Day of diagnosis: 2 0 | clinical  drain analysis  > bilirubin level in  drain: ____ mg/dl  CT / MRI  during operation | conservative  interventional  drain insertion  intraabdominal  PTCD  ERCP  with stenting  reoperation |
| Bilioma yes no  Day of diagnosis: 2 0 | CT / MRI  during operation | conservative  interventional  drain insertion  reoperation |
| Postoperative pancreatic yes no  fistula (POPF)  Grade (ISGPS) A B C  Day of diagnosis: 2 0 | clinical  drain analysis  > amylase level in  drain: ____ U/l  CT / MRI  during operation | conservative  interventional  drain insertion  reoperation |
| Abscess yes no  Day of diagnosis: 2 0 | CT / MRI  during operation | conservative  interventional  drain insertion  reoperation |
| Cholangitis yes no  Day of diagnosis: 2 0 | clinical  microbiology | antibiotics  Other: ________ |

**Visit 3 – POD 1 (Page 2 / 3)**

| **Morbidity** *(continued)* |  |  |
| --- | --- | --- |
| Stenosis yes no  of hepaticojejunostomy  Day of diagnosis: 2 0 | clinical  laboratory tests  CT / MRI  during operation | conservative  PTCD  ERCP  with stenting  reoperation |
| Surgical site yes no  infection  Day of disgnosis: 2 0 | clinical  microbiology | antibiotics  opening  surgery  other: ________  ______________ |
| Other complication: yes no  _________________  Day of diagnosis: 2 0 | diagnostic test:  ________________ | therapy:  ______________ |

**Preoperative laboratory tests**

Date of blood sample taking **2 0** *(dd/mm/yyyy)*

Hemoglobin __________ (g/dl) Leucocytes __________ (cells/ml)

Creatinine __________ (mg/dl) Albumin __________ (g/l)

ASAT __________ (U/l) ALAT __________ (U/l)

AP __________ (U/l) GGT __________ (U/l)

Bilirubin __________ (mg/dl) CRP __________ (mg/dl)

Quick value __________ (%)

**Visit 3 – POD 1 (Page 3 / 3)**

**Drain analysis**

Bilirubin level in wound drain: ______ mg/dl

No drain

**Mortality**

Death yes no

Date of death: **2 0** *(dd/mm/yyyy)*

Cause of death: ____________________________________________

Name of investigator in block letter: ___________________________________

Date: _____________ Signature: ___________________________

**Visit 4 – POD 3 (Page 1 / 3)**

Date of visit **2 0** *(dd/mm/yyyy)*

**Morbidity**

Has any complication occurred or yes no

is still active since the last visit?

**If yes,** please specify and complete the AE / SAE form for each event separately!

| **Complication** | **Diagnostic tests** | **Therapy** |
| --- | --- | --- |
| Postoperative yes no  insufficiency of  hepaticojejunostomy  Day of diagnosis: 2 0 | clinical  drain analysis  > bilirubin level in  drain: ____ mg/dl  CT / MRI  during operation | conservative  interventional  drain insertion  intraabdominal  PTCD  ERCP  with stenting  reoperation |
| Bilioma yes no  Day of diagnosis: 2 0 | CT / MRI  during operation | conservative  interventional  drain insertion  reoperation |
| Postoperative pancreatic yes no  fistula (POPF)  Grade (ISGPS) A B C  Day of diagnosis: 2 0 | clinical  drain analysis  > amylase level in  drain: ____ U/l  CT / MRI  during operation | conservative  interventional  drain insertion  reoperation |
| Abscess yes no  Day of diagnosis: 2 0 | CT / MRI  during operation | conservative  interventional  drain insertion  reoperation |
| Cholangitis yes no  Day of diagnosis: 2 0 | clinical  microbiology | antibiotics  Other: ________ |

**Visit 4 – POD 3 (Page 2 / 3)**

| **Morbidity** *(continued)* |  |  |
| --- | --- | --- |
| Stenosis yes no  of hepaticojejunostomy  Day of diagnosis: 2 0 | clinical  laboratory tests  CT / MRI  during operation | conservative  PTCD  ERCP  with stenting  reoperation |
| Surgical site yes no  infection  Day of disgnosis: 2 0 | clinical  microbiology | antibiotics  opening  surgery  other: ________  ______________ |
| Other complication: yes no  _________________  Day of diagnosis: 2 0 | diagnostic test:  ________________ | therapy:  ______________ |

**Preoperative laboratory tests**

Date of blood sample taking **2 0** *(dd/mm/yyyy)*

Hemoglobin __________ (g/dl) Leucocytes __________ (cells/ml)

Creatinine __________ (mg/dl) Albumin __________ (g/l)

ASAT __________ (U/l) ALAT __________ (U/l)

AP __________ (U/l) GGT __________ (U/l)

Bilirubin __________ (mg/dl) CRP __________ (mg/dl)

Quick value __________ (%)

**Visit 4 – POD 3 (Page 3 / 3)**

**Drain analysis**

Bilirubin level in wound drain: ______ mg/dl

No drain

**Mortality**

Death yes no

Date of death: **2 0** *(dd/mm/yyyy)*

Cause of death: ____________________________________________

Name of investigator in block letter: ___________________________________

Date: _____________ Signature: ___________________________

**Visit 5 – Day of discharge (Page 1 / 3)**

Date of visit: **2 0** *(dd/mm/yyyy)*

| **Final histopathological diagnosis**    Pancreatic ductal adenocarcinoma Distal bile duct carcinoma    Pancreatic neuroendocrine neoplasm Duodenal carcinoma  IPMN or cystic pancreatic neoplasm Ampullary carcinoma  Benigne bile duct stenosis Chronic pancreatitis  Other: __________________________________________________________  _________________________________________________ |
| --- |

| T | N | Positive lymph nodes (n) | Resected lymph nodes (n) | L | V | M | G |
| --- | --- | --- | --- | --- | --- | --- | --- |
|  |  |  |  |  |  |  |  |

**Intensive care unit stay**

Total days of ICU admission (1 day = overnight stay, not IMC ward): ____________

Readmission during postoperative stay: yes no

Cause of readmission: __________________________________

Day of readmission: **2 0** *(dd/mm/yyyy)*

**Krankenhausaufenthalt**

Day of admission: **2 0** *(dd/mm/yyyy)*

Day of discharge: **2 0** *(dd/mm/yyyy)*

Day of removal of drain placed at the hepaticojejunostomy: ____ postoperative day

Day of removal of last abdominal drain: ____ postoperative day

(including interventional drains placed postoperatively)

**Visit 5 – Day of discharge (Page 2 / 3)**

Date of visit **2 0** *(dd/mm/yyyy)*

**Morbidity**

Has any complication occurred or yes no

is still active since the last visit?

**If yes,** please specify and complete the AE / SAE form for each event separately!

| **Complication** | **Diagnostic tests** | **Therapy** |
| --- | --- | --- |
| Postoperative yes no  insufficiency of  hepaticojejunostomy  Day of diagnosis: 2 0 | clinical  drain analysis  > bilirubin level in  drain: ____ mg/dl  CT / MRI  during operation | conservative  interventional  drain insertion  intraabdominal  PTCD  ERCP  with stenting  reoperation |
| Bilioma yes no  Day of diagnosis: 2 0 | CT / MRI  during operation | conservative  interventional  drain insertion  reoperation |
| Postoperative pancreatic yes no  fistula (POPF)  Grade (ISGPS) A B C  Day of diagnosis: 2 0 | clinical  drain analysis  > amylase level in  drain: ____ U/l  CT / MRI  during operation | conservative  interventional  drain insertion  reoperation |
| Abscess yes no  Day of diagnosis: 2 0 | CT / MRI  during operation | conservative  interventional  drain insertion  reoperation |
| Cholangitis yes no  Day of diagnosis: 2 0 | clinical  microbiology | antibiotics  Other: ________ |

**Visit 5 – Day of discharge (Page 3 / 3)**

| **Morbidity** *(continued)* |  |  |
| --- | --- | --- |
| Stenosis yes no  of hepaticojejunostomy  Day of diagnosis: 2 0 | clinical  laboratory tests  CT / MRI  during operation | conservative  PTCD  ERCP  with stenting  reoperation |
| Surgical site yes no  infection  Day of disgnosis: 2 0 | clinical  microbiology | antibiotics  opening  surgery  other: ________  ______________ |
| Other complication: yes no  _________________  Day of diagnosis: 2 0 | diagnostic test:  ________________ | therapy:  ______________ |

**Mortality**

Death yes no

Date of death: **2 0** *(dd/mm/yyyy)*

Cause of death: ____________________________________________

Name of investigator in block letter: ___________________________________

Date: _____________ Signature: ___________________________

**Visit 6 – 3 months postoperative (Page 1 / 2)**

Date of visit **2 0** *(dd/mm/yyyy)*

**Morbidity**

Has any complication occurred or yes no

is still active since the last visit?

**If yes,** please specify and complete the AE / SAE form for each event separately!

| **Complication** | **Diagnostic tests** | **Therapy** |
| --- | --- | --- |
| Postoperative yes no  insufficiency of  hepaticojejunostomy  Day of diagnosis: 2 0 | clinical  drain analysis  > bilirubin level in  drain: ____ mg/dl  CT / MRI  during operation | conservative  interventional  drain insertion  intraabdominal  PTCD  ERCP  with stenting  reoperation |
| Bilioma yes no  Day of diagnosis: 2 0 | CT / MRI  during operation | conservative  interventional  drain insertion  reoperation |
| Postoperative pancreatic yes no  fistula (POPF)  Grade (ISGPS) A B C  Day of diagnosis: 2 0 | clinical  drain analysis  > amylase level in  drain: ____ U/l  CT / MRI  during operation | conservative  interventional  drain insertion  reoperation |
| Abscess yes no  Day of diagnosis: 2 0 | CT / MRI  during operation | conservative  interventional  drain insertion  reoperation |
| Cholangitis yes no  Day of diagnosis: 2 0 | clinical  microbiology | antibiotics  Other: ________ |

**Visit 6 – 3 months postoperative (Page 2 / 2)**

| **Morbidity** *(continued)* |  |  |
| --- | --- | --- |
| Stenosis yes no  of hepaticojejunostomy  Day of diagnosis: 2 0 | clinical  laboratory tests  CT / MRI  during operation | conservative  PTCD  ERCP  with stenting  reoperation |
| Surgical site yes no  Infection  Day of disgnosis: 2 0 | clinical  microbiology | antibiotics  opening  surgery  other: ________  ______________ |
| Other complication: yes no  _________________  Day of diagnosis: 2 0 | diagnostic test:  ________________ | therapy:  ______________ |

**Mortality**

Death yes no

Date of death: **2 0** *(dd/mm/yyyy)*

Cause of death: ____________________________________________

Name of investigator in block letter: ___________________________________

Date: _____________ Signature: ___________________________

**Visit 7 – 12 months postoperative (Page 1 / 2)**

Date of visit **2 0** *(dd/mm/yyyy)*

**Morbidity**

Has any complication occurred or yes no

is still active since the last visit?

**If yes,** please specify and complete the AE / SAE form for each event separately!

| **Complication** | **Diagnostic tests** | **Therapy** |
| --- | --- | --- |
| Postoperative yes no  insufficiency of  hepaticojejunostomy  Day of diagnosis: 2 0 | clinical  drain analysis  > bilirubin level in  drain: ____ mg/dl  CT / MRI  during operation | conservative  interventional  drain insertion  intraabdominal  PTCD  ERCP  with stenting  reoperation |
| Bilioma yes no  Day of diagnosis: 2 0 | CT / MRI  during operation | conservative  interventional  drain insertion  reoperation |
| Postoperative pancreatic yes no  fistula (POPF)  Grade (ISGPS) A B C  Day of diagnosis: 2 0 | clinical  drain analysis  > amylase level in  drain: ____ U/l  CT / MRI  during operation | conservative  interventional  drain insertion  reoperation |
| Abscess yes no  Day of diagnosis: 2 0 | CT / MRI  during operation | conservative  interventional  drain insertion  reoperation |
| Cholangitis yes no  Day of diagnosis: 2 0 | clinical  microbiology | antibiotics  Other: ________ |

**Visit 7 – 12 months postoperative (Page 2 / 2)**

| **Morbidity** *(continued)* |  |  |
| --- | --- | --- |
| Stenosis yes no  of hepaticojejunostomy  Day of diagnosis: 2 0 | clinical  laboratory tests  CT / MRI  during operation | conservative  PTCD  ERCP  with stenting  reoperation |
| Surgical site yes no  Infection  Day of disgnosis: 2 0 | clinical  microbiology | antibiotics  opening  surgery  other: ________  ______________ |
| Other complication: yes no  _________________  Day of diagnosis: 2 0 | diagnostic test:  ________________ | therapy:  ______________ |

**Mortality**

Death yes no

Date of death: **2 0** *(dd/mm/yyyy)*

Cause of death: ____________________________________________

Name of investigator in block letter: ___________________________________

Date: _____________ Signature: ___________________________

**Specific morbidity form (SMF) (Page 1 / 2)**

**(please use a separate form for each morbidity)**

Number of morbidity in this patient: _______ *(please number consecutively)*

Begin of morbidity **2 0** *(dd/mm/yyyy)*

End of morbidity **2 0** *(dd/mm/yyyy)*

**Symptoms / problems** *(Please specify)*

______________________________________________________________________

______________________________________________________________________

______________________________________________________________________

**Diagnostic measurements** *(Please specify)*

______________________________________________________________________

______________________________________________________________________

______________________________________________________________________

**Treatment** *(Please specify)*

______________________________________________________________________

______________________________________________________________________

_____________________________________________________________________

**Specific morbidity form (SMF) (Page 2 / 2)**

**(please use a separate form for each morbidity)**

**Classification according to Clavien-Dindo**

I II

IIIa IIIb

IV V

Morbidity ≥ III has to be reported by the attending physician or investigator to the principal investigator immediately within 24 h or not later than the next working day.

> Fax number: 09131/85-36595

> E-Mail: Maximilian.Brunner@uk-erlangen.de

**Sequelae**

Readmission necessary yes no

Prolonged hospitalisation yes no

Readmission to ICU yes no

**Outcome**

recovered completely recovered with sequelae

ongoing death

**Causality to trial intervention** (regarding performance of hepaticojejunostomy)

unrelated possibly related

probably related definitly related

not assessable

Name of investigator in block letter: ___________________________________

Date: _____________ Signature: ___________________________

**End of study**

Did the patient complete the study? yes no

If yes, date of study completion : **2 0** *(dd/mm/yyyy)*

If no, date of withdrawal / last contact: **2 0** *(dd/mm/yyyy)*

Reason for withdrawal:

______________________________________________________________________

______________________________________________________________________

**Investigators declaration**

I have read the data entered on all the CRF pages and certify that the data are complete

and accurate to the best of my knowledge.

Name of investigator in block letter: ___________________________________

Date: _____________ Signature: ___________________________
